# Psychological Profile and Visual Function in Charles Bonnet Syndrome: A Preliminary Cross-Sectional Study

**DOI:** 10.3390/healthcare14070885

**Published:** 2026-03-30

**Authors:** Emanuela Rellini, Valeria Silvestri, Margherita Guidobaldi, Simona Turco, Daniela Pia Rosaria Chieffo, Eliana Costanzo, Filippo Amore, Stefania Fortini

**Affiliations:** 1 National Centre of Service and Research for the Prevention of Blindness and Vision Rehabilitation of the Visually Impaired-IAPB ETS, Fondazione Policlinico Universitario Agostino Gemelli, IRCCS, 00168 Rome, Italy; v.silvestri@iapb.it (V.S.); m.guidobaldi@iapb.it (M.G.); s.turco@iapb.it (S.T.); danielapiarosaria.chieffo@policlinicogemelli.it (D.P.R.C.); f.amore@iapb.it (F.A.); s.fortini@iapb.it (S.F.); 2 Clinical Psychology Unit, Fondazione Policlinico Universitario Agostino Gemelli, IRCCS, 00168 Rome, Italy; 3 Ophthalmology Unit, Fondazione Policlinico Universitario Agostino Gemelli, IRCCS, 00168 Rome, Italy; 4 IRCCS—Fondazione Bietti, 00184 Rome, Italy; eliana.costanzo@fondazionebietti.it

**Keywords:** Charles Bonnet syndrome, low vision, anxiety, depression, psychological distress

## Abstract

**Purpose**: The purpose of this preliminary study was to investigate the prevalence of Charles Bonnet Syndrome (CBS) among patients attending the National Centre of Service and Research for the Prevention of Blindness and Vision Rehabilitation of the Visually Impaired, Rome, Italy. Furthermore, the research aimed to delineate the psychological profile of these individuals to determine whether significant differences exist compared with visually impaired patients who do not experience hallucinatory phenomena and to identify likely predictors. **Methods**: A preliminary cross-sectional analysis was conducted on a convenience sample of patients recruited between January 2025 and December 2025. Prevalence was calculated based on structured clinical interviews, while the psychological profile was assessed by comparing the CBS group with a control group (non-CBS) matched for visual acuity. Participants underwent comprehensive ophthalmological and psychological assessments, including best-corrected visual acuity (BCVA), reading acuity (RA), contrast sensitivity (CS), fixation stability, and retinal sensitivity (RS). Psychological status was evaluated using the Symptom Check List-90-Revised (SCL-90-R), the Patient Health Questionnaire (PHQ-9), and the Generalized Anxiety Disorder Questionnaire (GAD-7). Patients experiencing CBS were further interviewed regarding the specific characteristics and patterns of their hallucinations. The association between CBS and both psychological profiles and visual function parameters was evaluated using regression analysis. **Results**: Out of 385 individuals screened, 120 participants (58% women; mean age 55.4 ± 18.8 years) were included; CBS was detected in 19%. No significant differences were observed between participants with and without CBS in demographic variables or psychological questionnaire scores (*p* > 0.05). Mean SCL-90-R, PHQ-9, and GAD-7 scores indicated mild psychological distress, depression, and anxiety, with no significant group differences (*p* > 0.05). Using standard cut-off values, depressive and anxiety symptoms were prevalent in 65% and 88% of participants, respectively, but were not significantly associated with CBS in chi-square or logistic regression analyses (*p* > 0.05). Logistic regression analysis of SCL-90 scores showed that only anxiety was significantly associated with hallucination occurrence among the visually impaired participants (OR = 0.27; 95% CI = 0.08–0.87; *p* < 0.05). Among the visual function parameters, poorer RA in the worse eye was significantly associated with CBS (*p* < 0.05). **Conclusions**: This study confirms that CBS is a prevalent, yet frequently under-reported, condition within rehabilitation settings. While overall visual function did not differ significantly between patients with and without CBS, reduced reading acuity (RA) in the worse eye emerged as a potential specific risk factor. Characterizing the psychological profile of these patients is essential to differentiate the syndrome from psychiatric disorders and to develop tailored support pathways. Despite its preliminary nature, this research underscores the necessity of systematic screening to enhance clinical management and the emotional well-being of visually impaired individuals. Consequently, integrating psychological support into visual rehabilitation programs is vital to addressing the high prevalence of comorbid anxiety and depression.

## 1. Introduction

Vision impairment is a growing public health issue, particularly in aging populations. According to epidemiological data, approximately 16.7% of the general population has moderate vision impairment. The prevalence of visually impaired people rises to 28.8% among individuals over 65 years and 33.9% among those over 75 years. When considering both severe and moderate vision impairments, the prevalence increases to 18.6% in the general population, reaching 33.8% among people aged 65 or older and 41.9% in individuals over 75 years of age [1]. These estimates underline the increasing burden of visual disability and the need to address its broad consequences of reduced quality of life (QoL) and self-perceived well-being.

Among patients with chronic and degenerative ocular diseases—such as age-related macular degeneration (AMD), glaucoma, and diabetic retinopathy—there is a documented comorbidity with Charles Bonnet Syndrome (CBS). CBS is a neuro-ophthalmological condition characterized by complex visual hallucinations occurring in individuals with significant visual loss, in the absence of cognitive deterioration, psychiatric disorders, and primary neurological disease. The Swiss naturalist and philosopher Charles Bonnet first described CBS in 1769 by observing the phenomenon in his visually impaired grandfather. Since then, the syndrome has been widely documented but remains underdiagnosed and misunderstood.

The hallmark of CBS is the presence of vivid, recurring, and often intricate visual hallucinations, such as people, animals, or patterns, perceived by patients who are fully aware that these images are unreal. This preserved insight differentiates CBS from hallucinatory phenomena observed in psychiatric disorders, contributing to its classification as a condition arising from sensory deprivation. Multiple physiological hypotheses have been proposed to explain the etiology of CBS. One prevailing theory is that the syndrome results from cortical hyperexcitability in the visual association areas of the brain, particularly in the absence of adequate afferent stimulation from the retina. Functional Magnetic Resonance Imaging (fMRI) studies have demonstrated activation of the occipital and ventral temporal cortex during hallucinations in CBS patients, suggesting a form of “release phenomenon” whereby visual cortices become spontaneously active when deprived of normal input [2]. According to the deafferentation hypothesis, this activity is driven by molecular shifts—such as increased neurotransmitter release and altered GABAergic and NMDA receptor metabolism—which lower the threshold for cortical discharge. Recent evidence suggests that this hyperexcitability may represent a form of maladaptive neural plasticity, where the brain attempts to compensate for sensory loss by “filling in” the void with endogenous signals [3]. Another hypothesis points to maladaptive neuroplastic changes within the visual pathways, including a lack of inhibition in the lateral geniculate nucleus or disinhibition of feedback loops between the thalamus and cortex [4]. This state of chronic disinhibition means the visual cortex remains perpetually active even at rest, effectively limiting its capacity to respond to actual external stimuli while facilitating the emergence of spontaneous visual phenomena.

The reported prevalence of CBS varies significantly, with estimates ranging widely from 0.4% to as high as 63% [4]. This discrepancy is largely attributable to differing definitions of the syndrome, variability in study populations, and inconsistent methodologies [5,6]. In a recent meta-analysis, an overall prevalence of approximately 10.2% was identified among ophthalmic patients, suggesting that globally, about 47.2 million individuals may be affected [6].

Specific clinical populations show even higher rates: it is estimated that 15.8% of patients with age-related macular degeneration (AMD) experience CBS, a figure that rises to 31.3% in specialized low-vision rehabilitation centers. In patients with glaucoma, the prevalence is approximately 13.5% [7,8,9,10,11]. This variability is often compounded by a general lack of awareness among healthcare professionals and the use of non-standardized screening tools.

Furthermore, under-reporting remains a critical barrier to accurate epidemiology. Many patients are reluctant to disclose visual hallucinations for fear of being misdiagnosed with a psychiatric disorder or early-onset dementia. The stigma associated with “seeing things” often leads to “silent” suffering, resulting in delayed diagnosis and missed opportunities for support. Interestingly, while CBS is often a consequence of sensory loss, it has also been suggested as a potential risk factor for mental illness or a precursor to cognitive decline, a relationship that warrants further longitudinal investigation [4].

While some patients consider them amusing or neutral, others find them deeply distressing. Hallucinations can interfere with daily functioning and compromise mental health, leading to significant psychological consequences such as confusion, anxiety, anger, paranoia, and social withdrawal [12,13,14,15]. Additionally, patients have reported difficulties with sleep, diet, education, and work because of their visual experiences [16]. Approximately one-third of CBS patients reports distress and fear, particularly during the onset of symptoms or in the late stages of visual disease [12,13,14,16,17,18,19,20,21]. In a study by Jackson et al., patients diagnosed with glaucoma who experienced hallucinations were more likely to report depressive symptoms when compared with those without hallucinations [22,23]. On the other hand, other studies have not found a clear association between CBS and depression or mild cognitive impairment in patients with macular degeneration [24,25].

Moreover, anxiety has emerged as a prominent symptom in CBS, with up to 25% of patients having significant anxiety responses. Anxiety is characterized by excessive worry, somatic symptoms, and restlessness, and it can be amplified by the intrusive nature of hallucinations.

Interestingly, individuals with CBS often report lower scores on QoL measures than visually impaired individuals who do not experience hallucinations [16]. These psychological burdens raise essential questions regarding both the detection and management of CBS, particularly in the context of vision rehabilitation.

The primary objective of this study was to characterize the psychological profile of patients with CBS within a clinical rehabilitation sample. To our knowledge, this represents the first investigation in Italy to comprehensively assess emotional distress, anxiety, and psychological well-being in this specific population. A secondary aim was to determine if CBS is associated with distinct physiological markers of visual processing. To achieve this, we analyzed potential correlations between the syndrome and objective clinical measures, such as visual acuity, contrast sensitivity, and disease severity.

## 2. Methods

### 2.1. Setting and Participants

The present research was designed as an observational cross-sectional study. Participants were prospectively recruited from a convenience sample of visually impaired patients attending the National Centre of Services and Research for the Prevention of Blindness and Rehabilitation of the Visually Impaired—Fondazione Policlinico Universitario Agostino Gemelli (FPG) IRCCS—Rome, Italy, between 1 January 2025 and 31 December 2025. Patients were eligible if they were older than 18 years and were visually impaired or partially blind with a best-corrected visual acuity (BCVA) less than 0.4 LogMAR and/or a visual field less than 60% (≤20/50 Snellen and/or 60 degrees, according to the WHO standard).

Exclusion criteria were specifically defined to rule out confounding factors for visual hallucinations. These included: (1) neurological conditions such as Parkinson’s disease, Lewy body dementia, and history of stroke affecting the visual pathways; (2) severe cognitive impairment (MMSE < 24); (3) acute ophthalmological conditions other than stable low vision that could interfere with visual task performance, such as dense vitreous hemorrhage or acute corneal edema.

Data collection was performed by two sets of investigators: senior ophthalmologists [S.T. and F.A.] and orthoptists [V.S and M.G.], responsible for the clinical visual assessment, and clinical psychologists [E.R. and S.F], who administered and scored the psychological scales.

The study protocol was approved by the Ethical Committee/Institutional Review Board of the FPG—IRCCS of Rome, Italy [Prot N. 0001896/24; 5 December 2024]. The procedures used in this study adhere to the Tenets of the Declaration of Helsinki. All participants signed an informed consent before inclusion, and several measures were implemented to guarantee patient autonomy and data protection. Furthermore, all procedures were explained in accessible language to account for the visual impairment of the participants, ensuring that the “informed” nature of the consent was substantively met.

### 2.2. Procedure

1. All subjects who had access to our Low-Vision Centre underwent to both ophthalmologic and psychological evaluation in accordance with our clinical practice. After explaining the study aims, as per routine, patients underwent a clinical psychological interview that focused on emotional, cognitive and motivational prerequisites for undertaking vision rehabilitation. In addition, the presence or not of visual hallucinations was investigated. For subjects who reported the presence of visual hallucinations, specific questions were asked in order to better define clinical characteristics of the visual experience. Furthermore, screening questionnaires were administered to evaluate the mental health and psychological profile of the enrolled patients: the Symptom Check List (SCL-90-R), Patient Health Questionnaire-9 (PHQ-9) and Generalized Anxiety Disorder-7 (GAD-7). Participants completed the Italian standardized versions of the questionnaires. These versions have demonstrated robust psychometric properties within the Italian clinical and general population. Data from each participant were recorded during a single 2.5 to 3 h visit. In addition to the psychological questionnaire, the following ophthalmological measure was collected: best-corrected visual acuity (BCVA), evaluated by Early Treatment Diabetic Retinopathy Study (ETDRS) charts Precision Vision, Woodstock, IL, USA. ETDRS charts present a series of five letters of equal difficulty on each row, with standardized spacing between letters and rows, for a total of 14 lines (70 letters). BCVA was expressed in LogMAR values obtained at a distance of 4 m with the best refractive correction. BCVA was measured using a letter-by-letter scoring system for each eye separately based on the patients’ reading of all letters according to the following formula, which assigns a value of 0.02 log unit to each letter identified:
  LogMAR visual acuity score = 1.10 − 0.02 × TC,
  assuming that TC is equal to the total number of letters read correctly.
  Charts with different letter sequences were used for testing the right and left eyes. The better- and the worse-seeing eyes were identified as the eyes with the better and the worse visual acuity, respectively. To ensure consistency across the study population, the subjective refractive registration was performed following a standardized protocol starting with auto-refraction data and refining with ±0.50 spherical lenses, and then a ±0.50 Dcylinder was used to check if cylinder power was needed, testing at 90°, 180°, 45°, and 135° if necessary, while the non-tested eye was occluded. To minimize variability and sensory fatigue, a specific time of 15 min was strictly observed for each eye test.
2. Reading acuity (RA) was evaluated with the Minnesota Reading test (MNRead) charts at 25 cm with a +4.00 spherical dioptres (1×) reading lens in addition to the distance refractive correction. RA was noted in LogMAR values.
3. Contrast sensitivity (CS) was evaluated with Pelli–Robson charts at a 1 m distance, with the addition of +1.00 spherical dioptres to the distance refractive correction, in LogC notation.
4. Fixation stability was analyzed with an MP-1 microperimeter (Nidek Technologies, Padua, Italy). Patients were asked to fixate on a central target for approximately 30 s. The standard target was represented by a white cross with an extension of 1°. The data was quantified by calculating the Bivariate Contour Ellipse Area (BCEA) encompassing one standard deviation (1 − S.D., 68.2%) and was calculated in degrees^2^ using Steinman’s technique [26,27].
5. Retinal threshold sensitivity (RS) was evaluated with the MP-1 microperimeter (Nidek Technologies) expressed in decibels (dB), using an automatic macula program of 12°, −10 dB, a 4-2 threshold strategy and Goldmann size III stimuli.

### 2.3. Instrumentation

The methodological approach was based on the administration of the following tests: the Symptom Check List SCL-90-R, PHQ-9 and GAD-7. The SCL-90-R comprises 90 items that assess psychopathological or somatic disturbances on a 5-point scale ranging from 0 (absence of the symptom) to 4 (maximum disturbance). The 90 items are grouped into 9 scales labeled as: somatization (SOM for distress related to one’s body/physiological experiences); obsessive–compulsive behavior (OC, for intrusive thoughts and compulsive actions); interpersonal sensitivity (IS, describing self-perceived inadequacy/inferiority in relationships with others); depression (DEP, for low mood and decreased sense of meaning); anxiety (ANX, for anxious symptoms and experienced tensions); hostility (HOS, for aggressiveness towards others); phobic anxiety (PANX, for fears related to specific stimuli); paranoid ideation (PI, for projections of others and persecutory cognitions); psychoticism (PSY, for psychotic and schizophrenic behaviors); and sleeping disorder (SLED, alterations in the sleep–wake rhythm). Each of the 9 symptom dimensions were assessed with 6 to 13 items. The score on each dimension represents the mean score of all items of the dimension and directly reflects the severity of the mental health problem. Subscale scores ≥ 1 were suggestive of potential mental health issues. A higher factor score indicated more serious psychiatric symptoms problems. The global severity index (GSI) is a mean score of all 90 items [28,29,30].

The PHQ-9 is a tool used for screening, diagnosing, monitoring and determining the severity of depression [30]. The PHQ-9 consists of 9 items that correspond to the symptoms of major depression according to the Diagnostic and Statistical Manual of Mental Disorders IV (DSM-IV), also taken up in the DSM-5. Patient responses are marked from 0 to 3, with 0 representing “never” and 3 indicating “almost every day”; therefore, the PHQ-9 has a total score of 0–27. The questions of the PHQ-9 are simple and relate to depressive symptoms experienced in the last 2 weeks, which include anhedonia, dysphoria, sleep disorders, fatigue, eating disorders, low self-esteem, difficulty concentrating, and thoughts of suicide or homicide. If the score obtained in the questionnaire is greater than 5, it is important to refer the patient for a diagnostic investigation by a Clinical Psychologist, whereas if it is ≥15, a psychiatric evaluation is needed [31,32].

The GAD-7 is a screening questionnaire for anxiety disorders and allows for a reliable measure of the severity of the problem. The patient is asked how often, in the last two weeks, they have been bothered by each of the problems listed. The score is calculated by assigning 0, 1, 2 and 3 to the response categories of never, several days, more than half of the days, and every day. The total GAD-7 score for the seven items ranges from 0 to 21. Scores of 5, 10, and 15 represent reference points for mild, moderate and severe anxiety respectively. If the score obtained in the questionnaire is greater than 10, it is important to refer the patient for a diagnostic investigation by a Clinical Psychologist, whereas if it is ≥15, a psychiatric evaluation is needed [33,34].

### 2.4. Statistical Analysis

A preliminary sample size calculation was performed, suggesting that a larger cohort would be required for maximum statistical stringency. However, for the purpose of this study, a convenience sample of patients attending the center between 1 January 2025 and 31 December 2025 was analyzed. This approach allowed for the timely dissemination of clinical data while maintaining a robust recruitment window of 12 months. We believe the current sample size provides sufficient exploratory power to identify key trends and risk factors within this specific rehabilitation setting. Measurement data are presented as mean ± standard deviation (SD) or median (interquartile range), and count data are expressed as frequencies or percentages. To define the variables analyzed in each test, normality was assessed using the Shapiro–Wilk test. For continuous variables, a 2-tailed Student’s *t*-test was performed if assumptions of normality were achieved; otherwise, a non-parametric Mann–Whitney U test was used. For categorical variables, comparisons were made using the chi-square test. Linear regression modeling was performed to identify the variables predictive of CBS. The coding for dichotomous dependent variables for regression analyses was as follows: 0 (no_CBS) and 1 (CBS). Characteristics of patients with and without CBS were compared, and all factors found to be significant (*p* < 0.05) in the univariable analysis were subsequently included in the multivariable model to adjust for potential confounders. Statistical analyses were performed using Jamovi, Version 2.4 (The jamovi project, Sydney, Australia) [35], and statistical significance was set at *p* < 0.05.

## 3. Results

### 3.1. Demographic Data

**Figure 1:**
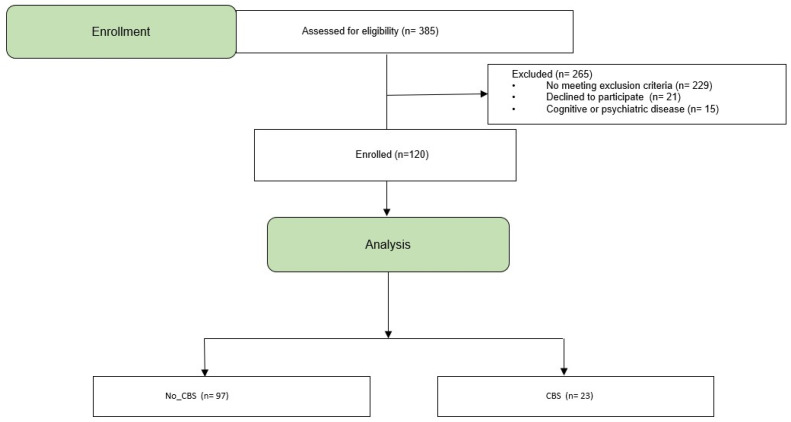
Flow diagram of the study. Three-hundred and eighty-five people were eligible to participate but 120 were enrolled and analyzed.

A total of 385 individuals were screened for eligibility (Figure 1). Of these, 172 participants were excluded due to total blindness or a BCVA > 0.4 LogMAR; 57 participants were younger than 18 years; and 21 declined participation because of poor general health. An additional 15 individuals (4%) were excluded because of a documented history of cognitive or psychiatric disorders.

The final sample consisted of 120 patients, including 70 women (58%).

The mean age was 55.4 ± 18.8 years (range 18–85). Thirty-three percent (n = 40 individuals) were ≤50 years, while 67% (n = 80 individuals) were older than 50 years. Secondary school and high school education were the most common educational levels (each n = 50, 42%).

Preliminary analyses were conducted to assess the comparability of the two groups. No statistically significant differences were observed between patients with CBS and those without CBS regarding demographic data (age and gender) or ocular clinical characteristics (type of pathology and visual acuity), ensuring that the cohorts were well-balanced for comparison (all *p* > 0.05).

Ocular diagnoses included age-related macular degeneration in (18%), high myopia (18%), retinitis pigmentosa (16%), Stargardt disease (15%), other inherited retinal diseases (11%), optic nerve disorders (11%), glaucoma (5%), and congenital ocular diseases (6%).

### 3.2. Prevalence and Characteristics of Visual Hallucinations

Visual hallucinations consistent with Charles Bonnet Syndrome (CBS) were reported by 23 participants (19%), while 97 (81%) denied such experiences.

Among those with CBS, 78% (18 out of 23) had communicated the hallucinations to someone, and only one had prior knowledge of the syndrome. The descriptive characteristics of hallucinations are reported in Table 1.

### 3.3. Depression

**Figure 2:**
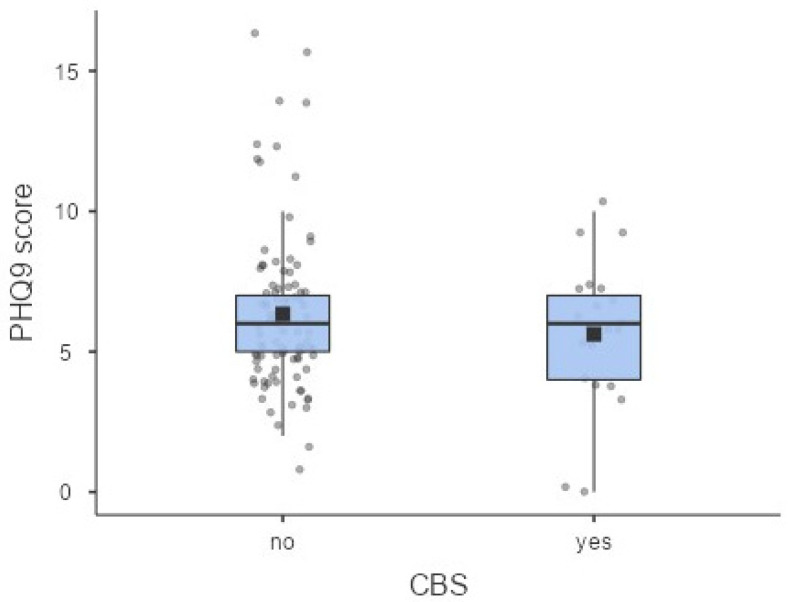
Box plots representing descriptive statistics for the PHQ-9 questionnaire between participants with CBS and those without. These plots summarize the central tendency and variability of depressive symptoms across the two groups. The black squares represent the mean values of the PHQ-9 score.

The average PHQ-9 score was 6.21 ± 2.81 and did not significantly differ between participants without CBS and those with CBS (6.34 ± 2.86 and 5.62 ± 2.56, respectively; *p* > 0.05) (Figure 2). Overall, 28% of participants (n = 33) had no depressive symptoms, 63% (n = 76) had mild depression, 7% (n = 9) had moderate depression, and 2% (n = 2) had moderately severe depression. Using a PHQ-9 score > 5 as a cut-off, 65% of patients were classified as having depressive symptoms. Among these individuals, 13% reported visual hallucinations consistent with CBS. Chi-square analysis showed no significant association between depressive symptoms and the presence of CBS (*p* > 0.05).

### 3.4. Anxiety

**Figure 3:**
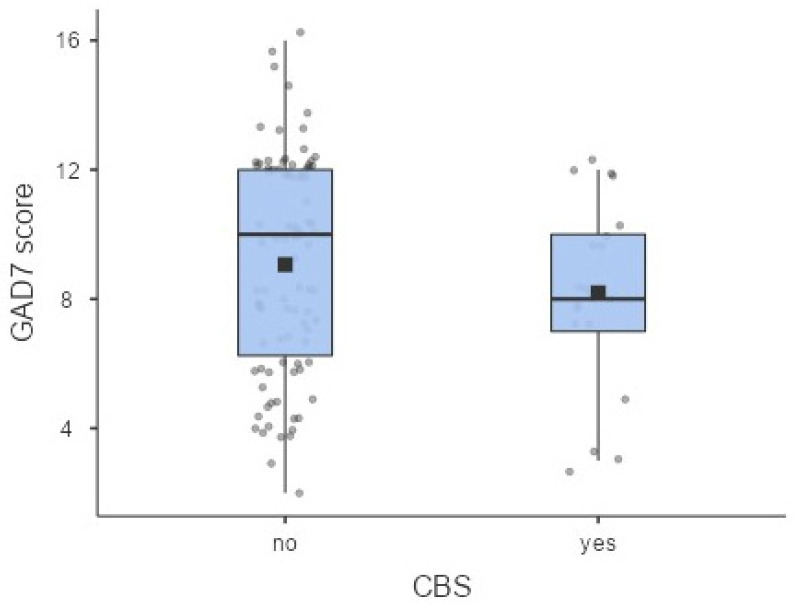
Box plots showing the distribution of GAD-7 scores for participants with and without Charles Bonnet Syndrome (CBS). These plots summarize the central tendency and variability of depressive symptoms across the two groups. The black squares represent the mean values of the GAD-7 score.

The mean GAD-7 score was 8.90 ± 3.21 with no significant differences between participants with and without CBS (8.12 ± 2.87 vs. 9.06 ± 3.27; *p* > 0.05) (Figure 3). Regarding severity, 12% (n = 14) reported no anxiety symptoms, 41% (n = 49) mild anxiety, 45% (n = 54) moderate anxiety, and 2% (n = 3) severe anxiety. Using a cut-off of >5, 88% of participants met the threshold for anxiety symptoms, and 83% of these individuals also had CBS. Chi-square analysis revealed no significant relationship between anxiety symptoms and CBS (*p* > 0.05). Logistic regression analysis confirmed that anxiety (yes vs. no) was not a significant predictor of CBS (OR 0.54; 95% CI 0.21–1.40; *p* > 0.05).

### 3.5. Quality of Life and Psychological Distress

Psychological distress was assessed using the SCL-90-1R questionnaire.

The average overall SCL-90-R score was 1.58 ± 0.37 with domain scores ranging from 1.32 to 1.86, indicating generally minor mental health and psychological well-being disorders in consideration of chronic and degenerative eye diseases causing visual impairment. No statistically significant differences in SCL-90-R domain scores were observed between participants with and without CBS (Table 2).

Moreover, we conducted a logistic regression analysis to identify psychopathological or somatic disturbances that predict hallucination occurrence. The results revealed that only anxiety (yes vs. no; OR = 0.27; 95% CI = 0.08–0.87; *p* < 0.05) had a statistically significant correlation with occurrence of hallucinatory experiences among the visually impaired enrolled.

### 3.6. Association Between CBS and Clinical Ophthalmological Characteristics

Clinical ophthalmological characteristics of the participants with and without CBS are shown in Table 3. No statistically significant differences were observed between the two groups regarding BCVA, RA and CS (*p* > 0.05) (Table 3).

However, univariable logistic regression analysis revealed a statistically significant association between the RA of the worse eye and the presence of CBS.

## 4. Discussion

The findings of the present study are broadly consistent with the existing body of research on Charles Bonnet Syndrome (CBS), particularly regarding its phenomenology and psychological correlates. The prevalence of CBS identified in this visually impaired Italian cohort was 19%. While some of the literature from specialized low-vision rehabilitation centers has reported rates as high as 31.3% [10], our findings align with recent meta-analytical data suggesting that patients in vision rehabilitation settings represent a high-risk group, with an average prevalence often exceeding 20% [5]. This variability in reported rates often reflects differences in screening methodologies and patient populations; however, our results confirm that when clinicians actively inquire about hallucinatory experiences, CBS emerges as a relatively common condition among patients with moderate to severe visual impairment.

The phenomenological profile of hallucinations observed among participants likewise mirrors established descriptions in the literature [2,4]. Patients reported vivid visual images—including figures, patterns, and complex scenes—while retaining intact insight regarding their unreality. This preserved insight remains one of the defining clinical hallmarks that distinguish CBS from hallucinations associated with psychotic or neurodegenerative disorders, as recently codified in the ICD-11 (9D56) [5]. In line with classical models, the results reinforce the interpretation of CBS as a sensory-deprivation phenomenon rooted in cortical hyperexcitability and spontaneous activation of visual association areas [5,30], a mechanism further supported by evidence highlighting deafferentation processes as a key driver of internally generated visual imagery [36].

The psychological evaluation in this study shows continuity with the emerging consensus that CBS patients often maintain stable emotional functioning despite the intrusive nature of their visual experiences. To the best of our knowledge, this study is the first to analyze the psychological profiles of visually impaired individuals with CBS using a comprehensive battery of validated instruments, specifically the SCL-90-R, PHQ-9, and GAD-7. Our results indicate relatively preserved mental health across the sample, a finding that stands in contrast to previous research [19,20,22] which identified strong associations between CBS and heightened anxiety or depressive symptoms, particularly in patients with glaucoma and late-stage AMD.

Among the domains of the Symptom Check List-90 (SCL-90), only anxiety showed a significant association with hallucination occurrence. The negative association observed (OR < 1) may indicate that visually impaired people experiencing anxiety are less likely to report hallucinatory experiences.

This divergence may indicate population-specific variations in emotional resilience, differences in expectations regarding visual disability, and the influence of contextual factors, such as access to multidisciplinary psychological support within the rehabilitation center. While some recent studies have identified correlations between negative affect, loneliness, and a more distressing appraisal of CBS symptoms [37], our findings highlight that a significant emotional burden is not a universal constant. The absence of a strong psychological burden in this cohort suggests that previous assumptions regarding the inherent emotional vulnerability of these individuals may not universally apply.

Interestingly, although univariate analyses initially suggested an association between CBS and specific psychological dimensions like anxiety, these factors did not retain statistical significance in the multivariable model. This crucial finding suggests that psychological distress in this population may be a comorbid feature of the severe visual impairment itself, rather than a direct driver or a specific consequence of the syndrome. This distinction—separating the psychological impact of sensory loss from the impact of CBS—is a significant nuance that warrants further longitudinal investigation [38].

A striking finding, consistent with the literature, is the low baseline awareness of CBS: only one patient had prior knowledge of the syndrome. This mirrors global trends showing weak awareness among both patients and healthcare providers, contributing to chronic under-reporting [5]. However, our study observed a high rate of disclosure (78%), which is significantly higher than the rates of concealment often reported due to fear of stigma. This may indicate that the multidisciplinary environment of an Italian rehabilitation center facilitates more open communication or that sociocultural factors support a more transparent discussion of perceptual experiences.

While confirming several well-established features, this study introduces an original contribution to the field: the identification of reading acuity (RA) in the worse eye as a statistically significant predictor of CBS. While CBS has traditionally been linked to generalized visual loss [5], the specific role of eye-specific reading acuity has rarely been isolated. This suggests that deficits in fine-detail processing—more than global loss of acuity—may trigger the compensatory cortical hyperexcitability underlying hallucination generation.

Despite these contributions, some limitations must be noted. The single-center design and the relatively small number of CBS cases for complex multivariable modeling limit the generalizability of the results. Furthermore, while we used validated psychological scales, the absence of qualitative interviews may have missed nuanced emotional reactions or coping strategies that are often central to the lived experience of the syndrome [37,38]. Furthermore, a limitation of this study is the lack of detailed data regarding the specific duration and dosage of pharmacological treatments for all participants.

In conclusion, this study—the first of its kind in Italy—underscores the importance of systematic screening and the integration of psychological support within visual rehabilitation programs. Addressing the persistent lack of awareness and focusing on specific functional markers like reading acuity will be essential for improving clinical management and the emotional well-being of visually impaired patients.

## 5. Conclusions

This study confirms that Charles Bonnet Syndrome (CBS) is a prevalent (19%) yet under-reported phenomenon among Italian visually impaired patients. Our findings provide a critical clinical distinction: CBS is not a primary psychiatric disorder—as demonstrated by the stable SCL-90-R, PHQ-9, and GAD-7 scores—but rather a sensory-deprivation phenomenon arising within a population already vulnerable to emotional distress linked to vision loss. The identification of poorer reading acuity in the worse eye as a significant predictor offers a novel, practical “early warning sign” for clinicians. We therefore advocate for a multidisciplinary rehabilitation model where systematic psychological screening and psychoeducational support are integrated. Such an approach is essential to reduce the stigma associated with hallucinations, prevent psychiatric misdiagnosis, and ensure a safe clinical environment for patient disclosure.

## Figures and Tables

**Table 1:** Table reporting characteristics in terms of pattern of hallucinatory phenomenon. People and animals, people and objects, flowers and objects were considered as complex scenes.

Hallucination Pattern	n (%)
People	7 (31)
Animal	4 (18)
Objects	3 (13)
Flowers	1 (4)
Geometric pattern	5 (22)
People and animals	1 (4)
People and objects	1 (4)
Flowers and objects	1 (4)

**Table 2:** Descriptive analysis of SCL-90 questionnaires of patients with and without Charles Bonnet Syndrome, reporting Cohen’s d effect size and 95% confidence intervals.

SCL-90 R	Items	no-CBS (n = 97)	CBS (n = 23)	t	*p*-Value	95% Confidence Interval (Inferior)	95% Confidence Interval (Superior)	Cohen’s d Effect Size
SOM	12	0.74 (±0.65)	0.68 (±0.74)	0.39	1.09	−41.26.00	6.12	15.16
OC	10	0.90 (±0.62)	0.71 (±0.68)	1.29	0.19	−16.52.00	8.03	50.03.00
IS	9	0.68 (±0.52)	0.60 (±0.83)	0.53	0.56	−32.20.00	5.52	22.1
DEP	13	0.94 (±0.68)	0.84 (±0.69)	1.02	0.53	−36.15.00	6.57	24.10.00
ANX	10	0.95 (±0.64)	0.71 (±0.47)	2.09	0.09	−6.55.00	8.47	65.22.00
HOS	6	0.55 (±0.60)	0.63 (±0.76)	−0.57	0.56	−63.21.00	3.29	−22.18.00
PANX	7	0.61 (±0.59)	0.55 (±0.56)	0.43	1.06	−35.20.00	5.32	16.51
PI	6	0.67 (±0.56)	0.47 (±0.55)	1.49	0.13	−10.44.00	7.36	57.50.00
PSY	10	0.34 (±0.36)	0.26 (±0.24)	1.04	0.29	−12.28.00	4.01	40.19.00
SLED	3	1.51 (±1.09)	0.83 (±1.01)	1.24	0.21	−31.18.00	13.44	49.07.00
GSI	86	0.70 (±0.43)	0.65 (±0.58)	1.09	1.09	−28.28.00	4.17	15.25

**Table 3:** Descriptive analysis of visual-functional assessment including eye diseases, visual field defects, best-corrected visual acuity, reading acuity and contrast sensitivity of patients with and without Charles Bonnet Syndrome.

Characteristic	no_CBS (n = 97)	CBS (n = 23)	*p*-Value
**Eye Disease, n (%)**			
AMD	19 (20)	3 (13)	
High Myopia	16 (17)	6 (26)	
Retinitis pigmentosa	16 (17)	3 (13)	
Stargardt Disease	15 (15)	3 (13)	
Inherited Retinal Disease	10 (10)	3 (13)	
Optic nerve disease	9 (9)	4 (18)	
Glaucoma	6 (6)	0	
Albinism	4 (4)	1 (4)	
ROP	2 (2)	0	
**Visual Field Defect, n (%)**			
Central	48 (49)	7 (30)	
Peripheral	27 (28)	5 (22)	
Mixed	22 (23)	11 (48)	
**Visual Acuity (ETDRS, LogMAR), mean (SD)**			
Better-seeing eye	0.63 (±0.37)	0.67 (±0.41)	0.62
Worse-seeing eye	0.89 (±0.40)	0.80 (±0.43)	0.43
**Reading Acuity (MNRead, LogMAR), mean (SD)**			
Eye with better LogMAR	0.44 (±0.34)	0.45 (±0.39)	0.52
Eye with worse LogMAR	0.69 (±0.41)	0.54 (±0.39)	0.16
**Contrast Sensitivity (Pelli-Robson, LogC), mean (SD)**			
Eye with better LogC	0.98 (±0.41)	0.92 (±0.40)	0.46
Eye with worse LogC	0.84 (±0.49)	0.83 (±0.42)	0.98

## Data Availability

The data presented in this study are available on request from the corresponding author. The data are not publicly available due to privacy and ethical restrictions.
